# A Novel Device-Free Counting Method Based on Channel Status Information

**DOI:** 10.3390/s18113981

**Published:** 2018-11-15

**Authors:** Junhuai Li, Pengjia Tu, Huaijun Wang, Kan Wang, Lei Yu

**Affiliations:** 1 School of Computer Science and Engineering, Xi’an University of Technology, Xi’an 710048, China; lijunhuai@xaut.edu.cn (J.L.); tpj1012@163.com (P.T.); wangkan@xaut.edu.cn (K.W.); yulei@xaut.edu.cn (L.Y.); 2 Shaanxi Key Laboratory for Network Computing and Security Technology, Xi’an 710048, China

**Keywords:** wavelet domain denoising, covariance matrix, spatial diversity, frequency diversity, robustness

## Abstract

Crowd counting is of significant importance for numerous applications, e.g., urban security, intelligent surveillance and crowd management. Existing crowd counting methods typically require specialized hardware deployment and strict operating conditions, thereby hindering their widespread application. To acquire a more effective crowd counting approach, a device-free counting method based on Channel Status Information (CSI) is proposed. The wavelet domain denoising is introduced to mitigate environment noise. Furthermore, the amplitude or phase covariance matrix is extracted as the eigenmatrix. Moreover, both the spatial diversity and frequency diversity are leveraged to improve detection robustness. At the same experimental environment, the accuracy of the proposed CSI-based method is compared with a renowned crowd counting one, i.e., Electronic Frog Eye: Counting Crowd Using WiFi (FCC). The experimental results reveal an accuracy improvement of 30% over FCC.

## 1. Introduction

In some overpopulated countries, the contradiction between the limited indoor space and large population is becoming increasingly prominent. Thus, it is of vital significance to implement crowd counting in public places, e.g., libraries, museums, shopping malls and college classrooms, which are with limited resources and strong mobility. In the meantime, it is a crucial and challenging task to acquire the human traffic or accurately calculate the population in some particular circumstances. Therefore, signal changes caused by human motion has been utilized to acquire crowd counting [1]. Since crowd counting can lead to an efficient utilization of space resources, it has been widely applied to intelligent surveillance, guided to tour, crowd management and urban security, etc.

Numerous crowd counting approaches have emerged over the past decades, e.g., video-based recognition, infrared-based induction, and non-image-based localization. However, these methods all require specialized hardware deployment and strict operating conditions which hinder their wide deployment. First, video-based recognition has the advantages of high-accuracy, rapidness and low-cost, and is applicable to various commercial and security fields. However, it is encountered by some constrains, e.g., many blind spots cannot be monitored and the environmental requirements are high [2]. Second, although infrared-based induction has also been widely applied in markets, subways and buses, it will be challenging to obtain a high counting accuracy, when the people flow becomes denser [3]. Third, non-image-based solutions typically require people to carry wearable devices. Unfortunately, it is impractical and expensive to distribute equipments to each individual in a public place, and is not feasible under an emergent event [4,5].

The legacy crowd counting methods not only consume manpower and materials, but also encounter the statistical error. In recent years, Wireless Sensor Network (WSN) technology has been gradually applied from theoretical research to industries. Besides, some network technologies, e.g., Wi-Fi., are also utilized in the field of crowd counting. The Received Signal Strength Indicator (RSSI) could realize crowd counting from the perspective of localization [6] and estimate human position in small areas or homes [7,8]. However, the RSSI is only applicable to the MAC layer information at the packet level, and its value will not remain stable with time fluctuation, and it meanwhile, does not provide sufficient recognition and robustness in complex indoor environments, thus the counting error will exist. Nowadays, some commercial ordinary wireless network cards (such as 802.11 a/g/n networks) can provide both amplitude and phase information on different subcarriers, utilizing the form of Channel State Information (CSI) by Orthogonal Frequency Division Multiplexing (OFDM). Different from RSSI, the CSI is a type of physical layer information and could obtain matrix of all subcarriers from transmit antennas to receive antennas [9]. More importantly, the CSI acquired by different subcarriers would exhibit distinct fading. These fading can be suppressd by the Multiple Input Multiple Output (MIMO) technique (such as IEEE 802.11n, 3GPP LTE, and mobile WiMAX systems), which can increase the capacity, range and reliability of wireless systems from the space dimension [10]. MIMO could enhance data throughput and transmission distance, without additional bandwidth and transmission power. Thus, CSI has both frequency diversity provided by OFDM and spatial diversity supplied by MIMO. As the specific RSSI at the physical layer, CSI is expected to enable more accurate and reliable detections, which has also attracted intensive attentions from both academic and industrial domains, and thus it would be a promising one in crowd counting.

The contradiction between limited indoor space and large population necessitates crowd number counting [1]. Meanwhile, people-counting also plays an significantly role in security monitoring and energy management for smart homes. We aim to find a baseline indicating the relationship between CSI and people number. In addition, it is worth noting that the widespread deployment of WiFi networks can also further boost our study. However, the same environment and conditions should be required (e.g., the same transmitter and receiver, as well as the same indoor environment) in this scenario, which has been widely considered in existing works [11,12].

The main contributions of our work are listed as follows:

- We present a device-free indoor people-counting method based on CSI. Since CSI is relatively stable in the static environment and sensitivity to human bodies, we leverage it to exactly achieve the crowd/people-counting.
- We utilize the linear transformation to obtain available phase information. In addition, the wavelet denoising is introduced to mitigate environment noises (e.g., indoor settings) and get relatively transparent CSI measurements. For multi-dimensional sample data, we extract the amplitude or phase covariance matrix as the eigenmatrix. Since covariance matrix can not only describe multi-dimensional data, but the diagonal elements represent the variance of each dimension.
- We attempt to combine both space diversity provided by MIMO and frequency diversity supplied by OFDM subcarriers, to enable more robust and accurate counting.
- We validate a monotonic relationship that lies between the CSI variations and crowd size. In addition, we utilize the percentage in the extension covariance matrix of amplitude and phase as baseline that estimate this relationship. Meanwhile, we compare the differences and similarities between phase and amplitude information in crowd counting.

## 2. Related Works

CSI can evaluate the channel information on each subcarrier, and characterize the frequency selective fading characteristics of Wi-Fi channels. In addition, CSI contains the amplitude and phase information on each subcarrier, thereby enhancing richer frequency domain information. Next, we summarize the existing works of the CSI form the aspect of phase and amplitude.

The amplitude-based detection. An amplitude-based scheme has attracted more attention in recent years since it is sensitive to adjacent humans, while unrelated background noise remains relatively stable. In this context, Xiao et al. [13] proposed a CSI feature extraction model based on the first and second largest eigenvalues of the Pearson correlation matrix of CSI amplitude, to detect the presence of humans. The existence detection is performed by outlier identification using normal features of density-based clustering. In [14], a monotonic function was proposed to depict the relationship between the crowd number and CSI amplitude variation. The function can be leveraged by a new dilated CSI matrix of the percentage of nonzero elements and the Grey Verhulst Model. In [15], a system was proposed to capture the variance between CSI amplitude of each subcarrier as features and uses Hidden Markov Model (HMM) to detect the human movement with different speed followed by a speed independent device free entity detection. In E-eyes [16], the distribution of CSI amplitude was utilized to classify the activities in entire home environment and identifies activities by calculating the similarity of each CSI segment and pre-structured activity files. Han et al. [17] designed a device-independent passive fall detection system using time stability and frequency diversity of CSI amplitude, in view of the fact the static human body is independent of the time domain variation of wireless signals. Experimental results showed that Wi-Fall can achieve a detection accuracy of 87%.

The Amplitude and Phase-based detection. Despite the fact that quite a few works have studied CSI for device-free detection, most previous studies only utilized amplitude information but not phase information. However, in [18], it was found that the CSI phase information is more sensitive to the detection process. Therefore, both the amplitude and phase information are utilized to calculate the maximum eigenvalues of Pearson correlation matrices, in order to compose a two-dimensional feature to infer the existence of a moving human body. This paper further employed an SVM machine learning model to achieve human moving detection. In [19], a new feature with the coefficient of phase variation was defined. The coefficient of phase variation is the ratio of the standard deviation to the mean of the CSI phase, in which human movement is detected when the averaged ratio is set within a predefined confidence interval. Qian et al. [20] proposed a device-free PAssive detection of moving humans with Dynamic Speed (PADS). The features are extracted from the covariance matrices of both CSI amplitude and phase, then, the eigenvalues of both matrices are calculated; maximum and second maximum eigenvalue of each matrix to form a four-tuple of features are selected; meanwhile, the SVM classifier to detect humans is selected. Cheng et al. [21] proposed an enhanced people-counting system with the DNN model as the classifier. In particular, a feature space expansion scheme is presented to enhance the DNN model. Nevertheless, this paper only showed the CSI is related to the crowd counting in indoor environment, without illustrating phase is more sensitive and explaining between the relationship CSI and the number of crowd.

Although there are prior works that utilize the CSI amplitude and phase information to conduct detection of moving humans, they typically ignore the relationship between the variation of CSI amplitude or phase and the number of moving people. In our work, an explicit relationship description is concentrated. In addition, we should also seek a baseline to indicate the relationship. Meanwhile, to further illustrate that the phase information is not only available, but is also more sensitive to the environment factors than amplitude information. Therefore, we attempt to respectively utilize the amplitude and phase information to achieve the counting, and exploit the frequency diversity and spatial diversity of CSI to obtain more accurate and robust counting result.

## 3. Crowd Counting Based on CSI

### 3.1. Architecture

**Figure 1:**
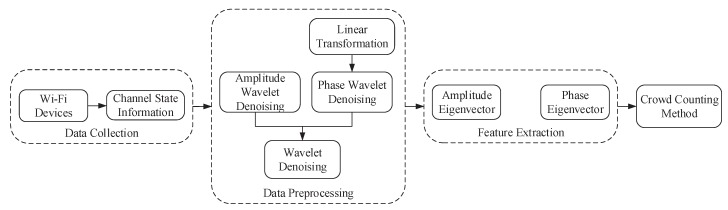
System architecture.

Figure 1 shows the system architecture of this paper, and the system architecture consists of four stages: (1) data collection; (2) data preprocessing; (3) feature extraction and (4) learning counting method. To verify the validity of the architecture, we perform a series of experiments to examine the relationship between CSI changes and the number of moving people.

First, we obtain CSI observations by different numbers of volunteers and, then, filter out both phase and amplitude noise from original CSI observations through wavelet denoising. It is worth noting that the phase information needs linear transformation.

Second, following data preprocessing, we further extract the covariance matrix as the eigenvector from both the amplitude and phase information, respectively.

Finally, since multiple transmit and receiver antennas combine into different streams, CSI consists of these streams, which constitute the spatial diversity of CSI. Meanwhile, the multipath effect exists indoors, which makes different subcarriers provide frequency diversity for CSI. This will also make the covariance characteristic vectors more reliable. Therefore, these works will enhance robustness, accuracy, and reliability of our method.

### 3.2. Data Preprocessing

Although standard wireless network cards provide RSSI information, the RSSI is only a rough estimate of the wireless channel, and it does not involve a specific number of antennas and subcarriers. Nowadays, some common IEEE 802.11n standard commercial wireless network cards have begun to emerge, which could provide detailed amplitude and phase information on different subcarriers in the form of CSI. The CSI is a type of physical layer information on the subcarrier scale, referring to the channel characteristics of a communication link. This type of information describes how the signal passes through the air from transmitter to receiver, and reflects the fading factor of signal on each transmission path, environmental degradation, signal scattering, and power attenuation with distance, etc.

In the narrow-band flat fading channel, the OFDM system in the frequency domain is modelled as

(1) y=Hx+N,

where *y*, *x*, *H* and *N* are respectively denoted as the receive vector, the transmit vector, the channel matrix and the additive white Gaussian noise (AWGN) vector.

Next, the CSI message, which represents the channel response of multiple subcarriers, is divided into 30 groups. Hence, the CSI value with N=30 groups collected at the receive that can be represented as

(2) $$\begin{array}{c} H(f_{i})=∥H(f_{i})∥e^{{jsin}^{\left(∠H(f_{i})\right)}},\\ i\in [1,30], \end{array}$$

where $H(f_{i})$ is the CSI at the subcarrier with the carrier frequency $f_{i}$, $∠H(f_{i})$ denotes its amplitude value and $∥H(f_{i})∥$ denotes its phase value.

Environmental factors (such as temperature, lighting and room settings) might appear with outliers in the CSI measurements, and these outliers would affect the detection performance to some extent. Thus, the measurements of amplitude and phase of CSI will be the basic input for our method, which would be through a wavelet transform denoising process beforehand. The wavelet transform denoising removes the observation value with larger deviations and meanwhile restore original signals as much as possible.

Given the original signal $C_{\varphi }$ , φ(t) constitutes the fundamental wavelet, i.e., if φ(t) meets Equation (3)

(3) $$C_{\varphi }=2\pi \int_{-\infty }^{+\infty }\frac{\varphi {\left(w\right)}^{2}}{\left|w\right|}dw<\infty ,$$

then the continuous wavelet transform f(t) can be written as

(4) $$\begin{array}{c} W_{f}\left(a,b\right)=\frac{1}{\sqrt{C_{\varphi }}}-\frac{1}{\sqrt{\left|a\right|}}\int_{-\infty }^{+\infty }f\left(t\right)\bar{\varphi }\left(\frac{t-b}{a}\right)dt,\\ a,b\in R,a\neq 0, \end{array}$$

where $\bar{\varphi }$ denotes the conjugate of φ , and *R* represents a real number set.

The associated inverse transformation formula f(t) can be written as

(5) $$f\left(t\right)=\frac{1}{\sqrt{C_{\varphi }}}\int_{-\infty }^{+\infty }db\int_{-\infty }^{+\infty }W_{f}\left(a,b\right){\left|a\right|}^{-\frac{1}{2}}\varphi \left(\frac{t-b}{a}\right)\frac{da}{a^{2}}.$$

**Figure 2:**
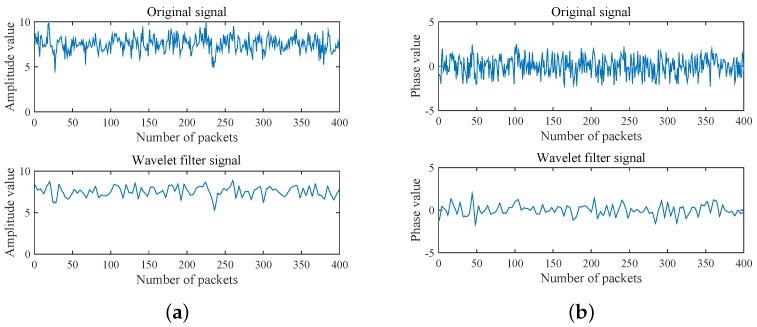
Comparison between the original and the filtered signal. (**a**) amplitude; (**b**) phase.

In the following, Figure 2 illustrates the original CSI amplitude and phase as well as the filtered signal, respectively. Among them, Symlet is a discrete wavelet function, which can reduce signal phase distortion to a certain extent. In this paper, the dataset is discrete and the data volume is relatively small. In addition, to ensure the integrity and readability of denoising CSI data, system wavelet is selected and wavelet scale is set to 2 in this work. From Figure 2, it is observed that the graph curve becomes smoother after the signal is filtered by the wavelet, and the frequent random fluctuations are filtered out. Thus, wavelet denoising can effectively filter out interference signals. It can be also revealed that the denoised signal retains the basic characteristics of the original one as well.

### 3.3. Feature Extraction

A suitable feature plays a key role. Nowadays, various statistical features [22,23,24] have been exploited for detection, such as the variance, mean, std, max-min. Nevertheless, these measures can only describe one-dimensional data (i.e., scalar), which necessitates the introduction of amplitude and phase covariance matrix as a feature one to enable counting in the multi-dimensional data. Then, the corresponding feature matrix can be represented as,

(6) $$\begin{array}{cc} a(i,j) & =cov(∠H(i),∠H(j)),\\ b(i,j) & =cov(∥H(f_{i})∥,∥H(f_{j})∥), \end{array}$$

where ∠H(i) , $∥H(f_{i})∥$ are the phase and amplitude information, respectively.

In the following, *A* and *B* are utilized to denote the amplitude and phase covariance matrix, respectively, i.e.,

(7) $$\begin{array}{cc} A & ={\left[a(i,j)\right]}_{N*N},\\ B & ={\left[b(i,j)\right]}_{N*N}, \end{array}$$

where a(i,j) and b(i,j) denote the phase and information covariance between vectors *i* and *j*, respectively.

**Figure 3:**
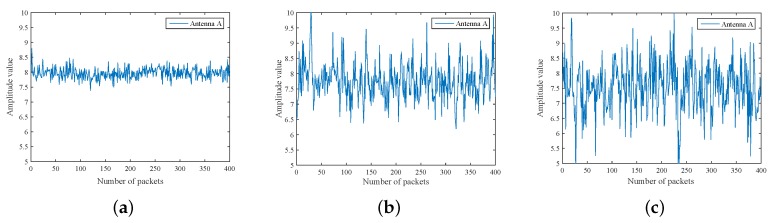
CSI amplitude changes with the people number on one antenna. (**a**) zero people; (**b**) two people; (**c**) four people.

**Figure 4:**
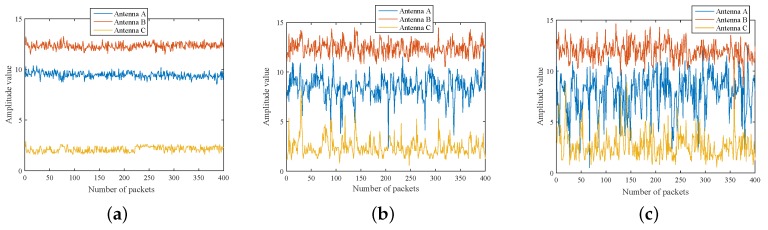
CSI amplitude changes with the people number on three antennas. (**a**) zero people; (**b**) two people; (**c**) four people.

Both Figure 3 and Figure 4 show the variation of CSI amplitude values with different numbers of moving people. Herein, Figure 3 demonstrates the CSI changes with one antenna when 0, 2, 4 people are walking, respectively, and Figure 4 displays the change with three antennas. The X-axis denotes the package index, while the Y-axis denotes the CSI amplitude value. For both cases, it can be clearly observed that the different degree of change of the CSI amplitude with the number of people increases. In addition, the amplitude varies sharply with the increase of packet number instantaneous. Therefore, the amplitude information can be considered as means of the crowd counting.

**Figure 5:**
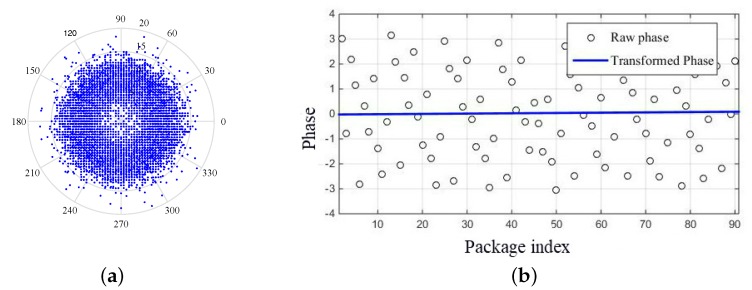
Phase before and after linear transformation. (**a**) random raw phase measurements; (**b**) phase after linear transformation.

Since there is a random noise and unsynchronized time clock, the raw phase information is unavailable, as shown in Figure 5a. However, most existing works also involve the phase information [18,19,25,26]. One of the most important reasons is that the phase information is more sensitive. In our work, in order to achieve people-counting through the phase information, we employ a linear transformation to remove random noise from the raw phase values. The measured phase $\hat{\phi_{i}}$ for the *i*th subcarrier can be expressed as

(8) $$\hat{\phi_{i}}=\phi_{i}+2\pi k_{i}\Delta t+2\pi k_{i}\Delta w,$$

where $\phi_{i}$ is the raw phase. $2\pi k_{i}\Delta t$ and $2\pi k_{i}\Delta w$ are the unknown phase shifts caused by the clock offset *t* and frequency difference *w*, respectively.

To remove the impact of random noise, the linear transformation can be written as

(9) $$\bar{\phi }=\hat{\phi_{i}}-\alpha k_{i}-\beta ,$$

where α and β denote intuitively the slope and offset of phase change over all subcarriers, respectively, $\bar{\phi }$ is the random phase offset which has been removed. Figure 5b illustrates an example of the phase after transformation, which is stably distributed.

### 3.4. Crowd Counting Algorithm

Our work aims to achieve crowd counting indoors (i.e., laboratory, corridor and office) settings at the Xi’an University of Technology, that is, the relationship between the number of moving people and CSI amplitude and phase variances is found, respectively. In PEM, presented in [14], the percentage of non-zero Element, can demonstrate the crowd size. PEM can adaptively reflect the relationship between CSI and the people number. In other words, it can intuitively represent different numbers of people. Neverthless, the FCC system [14] only considers amplitude information and directly uses the raw amplitude, i.e., the CSI phase information is ignored. Therefore, we propose to further improve the FCC system. In particular, it proposes to mitigate the environmental noise by wavelet denoising, extract CSI amplitude and phase covariance matrix as feature matrices and convert them into two-dimensional matrix, and expand the two-dimensional matrix using an algorithm and calculate the non-zero elements percentage of dilatation matrix. The idea of the expansion matrix method is that when a point expands to a certain size in the form of a circle, it will coincide with other points, and the size of these coincidence areas is contrary to the change of CSI strength. It should be noted that, our proposed method is with small amount of calculations and the data amount is less, leading to a counting time in the magnitude order of milliseconds. In Algorithm 1, C[i][j] represents the processed two-dimensional matrix (S×P), with and being the number of subcarriers and packets, respectively. $C_{min}$ and $C_{max}$ are the maximum and minimum value of covariance matrix, respectively, and *D* is the dilatation coefficient and *q* is percentage.

The aforementioned crowd counting approach can be listed as follows:

- We adopt wavelet transforms to remove the random noise, and then extract both the CSI amplitude and phase covariance matrix as the feature vector.
- All elements in the CSI amplitude or phase covariance matrix $M_{0}$ are initialized to “0”, and each CSI value C[i][j] is converted into integer *k* by $k=\left\lceil \frac{C\left[i\right]\left[j\right]-C_{min}}{C_{max}-C_{min}}\cdot \left(R-1\right)\right\rceil$. Then, the elements in row *k* and column *j* of $M_{0}$ is set to be “1”.
- The elements around *D* are set to “1”, which is called matrix expansion. The size of the expansion is related to the *D*. After dilation, the $M_{0}$ is transformed into matrix *M*. The matrix *M* is occupied by more “1”, and usually comes along with the CSI changes drastically.
- The number of “1” in the matrix *M* is counted, and the percentage of non-zero elements in the matrix *M* of each subcarrier is calculated by q=q/(P×S). This percentage is a guideline that the relationship between the CSI and the number of moving people.

**Algorithm 1** Crowd Counting based on Channel State Information**Require:** Sample Data $\left(\left[H_{1},H_{2},\ldots ,H_{k}\right],k\in \left(1,30\right)\right)$, Matrix Resolution $\left(R\right)$, Dilatation Coefficient   $\left(D\right)$, Number of Subcarrier $\left(S\right)$, Number of Packets $\left(P\right)$, Covariance Matrix $\left(C(i,j)\right)$, Maximum   Value of Covariance $\left(C_{max}\right)$, Minimum Value of Covariance $\left(C_{min}\right)$, Expansion Matrix $\left(M\right)$ **Ensure:** Percentage of Element $\left(q\right)$
1:the covariance matrix is extracted from wavelet transformed data2:**for**i=1 to S **do**3:    the integet *k* is calculated4:    the matrix *M* is dilated // the element in row *k* and column *j* is set to be “1”5:    **for** *u* = −D to *D* **do**6:        **for** *v* = −D to *D* **do**7:           the matrix *M* is dilated // the element in a radius of *D* is set to be “1”8:        **end for**9:    **end for**10:**end for**11:**for**l=1 to *P* **do**12:    **for** h=1 to *S* **do**13:        *M* = *M*$\left(1,h\right)$ // the percentage of the matrix *M* of each subcarrier14:        *q* = *q*/$\left(P\times S\right)$ // the percentage is calculated15:    **end for**16:**end for**


### 3.5. Leveraging Space Diversity

Nowadays, multiple antennas are adopted in more and more popular MIMO communication systems, since the signal strength variability can be reduced based on small-scale fading compensation. In an MIMO system, both the transmitter and receiver have multiple antennas, and each combination of receiving and transmitting antennas can be regarded as a stream. CSI consists of these streams. In addition, each stream implies a spatial choice. That is, these streams provide spatial diversity for CSI. Due to different propagation paths of diverse streams in the indoor environment, the CSI received by different antennas are differentiated. Therefore, all streams $H(f_{i})$ are with p×q dimensions and can be represented as:

(10) $$H\left(f_{i}\right)=\left[\begin{array}{cccc} h_{11} & h_{12} & \cdots & h_{1q}\\ h_{21} & h_{22} & \cdots & h_{2q}\\ \vdots & \cdots & \cdots & \vdots \\ h_{p1} & h_{p2} & \cdots & h_{pq} \end{array}\right],$$

where $h_{pq}$ is complex numbers representing the amplitude and phase of each subcarrier for an antenna, and *p*, *q* indicate the number of transimit and receive antennas, respectively.

**Figure 6:**
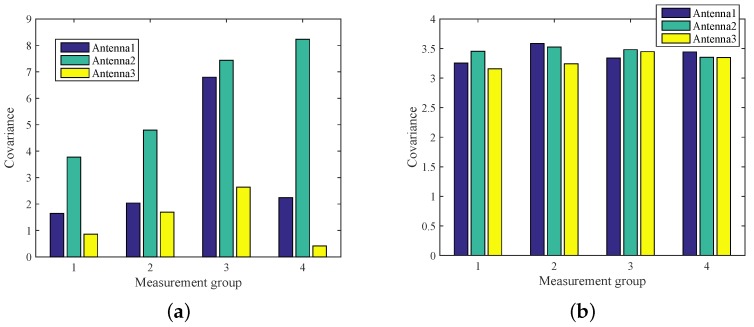
Antenna diversity: (**a**) antenna diversity of amplitude features; (**b**) antenna diversity of phase features.

As such, without enhancing the bandwidth and transmission power, multiple antennas can improve the reliability of spatial diversity [27,28]. Figure 6a shows the amplitude characteristic distribution of different numbers of people, while the phase characteristic distribution is displayed in Figure 6b. It can be observed that the features distribution of amplitude and phase do vary over different antennas. In addition, the phase features of all antennas remain relatively stable, but the amplitude features exist significant differences. Meanwhile, the feature variation is also different, whether in amplitude or phase. In this work, we exploit multiple antennas to improve the accuracy and robustness of counting a crowd. Therefore, the antenna with less variation should be selected when counting different people. If the antenna which great variation is selected, that is, the antenna is utilized mistakenly.

## 4. Experiment Results and Analysis

### 4.1. Experiment Setup

**Figure 7:**
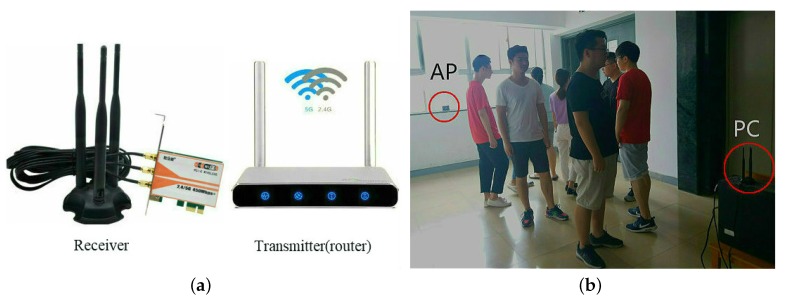
The experiments equipment and scenes. (**a**) receiver and router; (**b**) experiment scene.

To evaluate the performance of proposed approach, a real experiment platform is built at the elevator. We utilize the AP-link wireless router with two-antennas as transmitter and the PC with three-antennas as receiver, which form monitoring area. As illustrated in Figure 7a,b, all volunteers randomly walk in the monitoring area. In addition, considering the influence of different moving speed on counting accuracy, as such, volunteers have the same speed motion in this paper.

Since CSI information is highly sensitive to environmental factors. If too much people closely walk with each other in the monitoring area, it inevitably appears signal occlusion. Therefore, in this paper, the sensing range is 25 m${}^{2}$ of the elevator mouth, both AP and PC are placed at the height of 1.5 m, the distance between them is 5 m. Different volunteers scattered move within the sensing range, the transmitter continuously sends packets to the receiver, and the CSI information will be detected. It’s worth mentioning that these people try not to hide other people while walking. The 4 min data is collected at each sample point and 400 stable data packets are selected in the intermediate state, which will be experimental samples.

### 4.2. Relation Between Population and Amplitude (or Phase)

**Figure 8:**
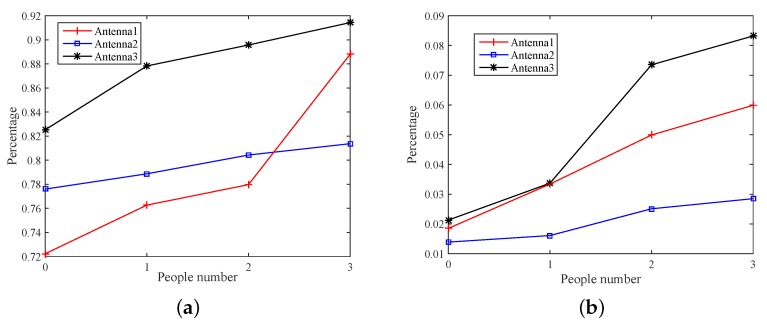
The relationship between the number of people and the percentage at the different antennas. (**a**) amplitude; (**b**) phase.

Figure 8 shows the relationship between the percentage and the number of moving people on different antennas. It is observed that a monotonous relationship between them. Among them, Figure 8a reveals the percentage of CSI amplitude when 0, 1, 2 and 3 people are walking, and Figure 8b shows the percentage change in CSI phase. Although the monotonic relation exists both in amplitude and phase information, the curve track of is different. However, it can be observed that the trajectory of phase information on different antennas is more concentrated, and the curve changes more dramatically when describing the monotonic relationship.

**Figure 9:**
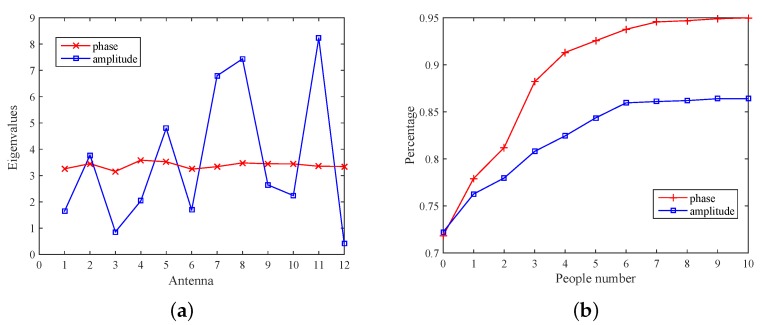
Comparison of amplitude and phase. (**a**) Comparison of mean in eigenvalue; (**b**) the relationship between percentage and the number of people.

In this work, the CSI phase or amplitude covariance matrix is extracted to achieve crowd counting. Figure 9a compares the variance of each dimension of the amplitude and phase, that is the diagonal elements of covariance matrix. It can be observed that the amplitude information is with a large fluctuation, while the phase information fluctuate is relatively stable. Figure 9b shows the percentage of amplitude and phase crowding count, where the percentage of amplitude information increase slowly from the sixth person and phase from the ninth person. Thus, the phase information is more reliable and sensitive to environmental changes and can detect more people.

### 4.3. Impact of Dilatation Coefficient

**Figure 10:**
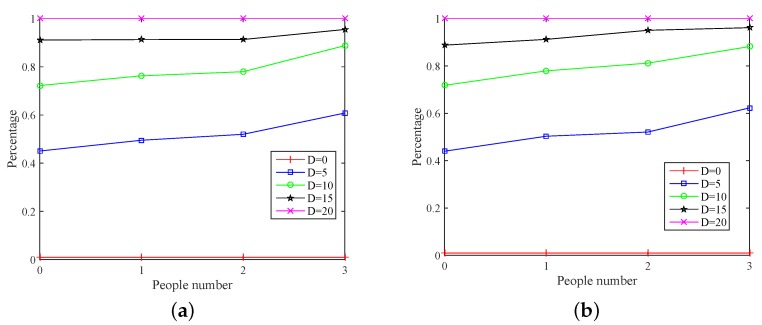
The impact on dilatation coefficient. (**a**) amplitude; (**b**) phase.

The dilatation coefficient is a scaling factor, which used to expand the percentage. We random choose integer 0–20 as scale factor, and this factor is not affected by the environmental change. Figure 10 shows the impact of dilatation coefficient. It can be seen that the percentage increases with the growth of dilatation coefficient, when the matrix resolution *R* is constant. Among them, the percentage reveals the relationship between the number of people and CSI variations. Although there is great difference in the percentage when the number of people is different, it would not induce detection errors. Figure 10a,b shows the variation on the percentage of amplitude and phase (under different dilatation coefficient *D*), respectively.

When D=0, the percentage remains constant with the increase in the number of people, since each column has only one non-zero element before dilatation. When D=20, the percentage not only keeps constant, but tends to 100%. As such, the dilatation coefficient D=20 is the maximum scale factor in this paper. Thus, if *D* is excessively either high or low, then it will affect the detection results. However, when *D* is equal to 5, 10 and 15, although there exists a monotone increase between percentage and number of people, the curve track is different. Therefore, the different dilation coefficients will bring different experimental results. Many experimental results show that D=10 is optimal value selection. Therefore, in our work, D=10 is set as the dilation coefficient.

### 4.4. Comparison with Existing Approaches

Table 1 compares the number of people identified by FCC system with our method. The percentage is used to indicate the number of people in the sensing area, and The percentage is saturated in the table. In addition, it is worth noting that the comparison result is performed in the same environment and the same setting conditions (i.e., same position of transmitters and receivers, same volunteers and equipment) [29]. For phase information, the percentage reached saturation of our method from the seventh person, which FCC system from the fourth person. The percentage reached saturation means that the method can detect so many people at most. In other words, although the percentage increases as the number of moving people grows, it would be saturated when the crowd density reaches a certain threshold. Therefore, for the percentage at saturation, our method is 42% higher than the FCC system, with three more people identified. A similar case occurs at the amplitude.

## 5. Conclusions

Crowd counting plays a key role in many applications, but existing counting methods usually require specialized hardware deployment and strict use of conditions which hinder their wide deployment. Therefore, a Device-Free Indoor people-counting Method based on Channel Status Information is proposed in this paper. The experimental results show that a monotonic relationship lies between the CSI variations and crowd size in indoor environments (i.e., laboratory, corridor and office). Meanwhile, from the similarities and differences of amplitude and phase changes that CSI phase has higher detection accuracy and sensitivity. Comparing the proposed method with FCC systems in the same experimental environment, it can be observed that the proposed method can detect more people and have higher counting accuracy, regardless of either the amplitude or phase covariance matrix as the feature vector. In the next step, a method should be proposed to accurately estimate the population in the special environment with more people. Moreover, the more accurate method will be studied when the target has different speeds.

## Figures and Tables

**Table 1:** Comparison FCC with our method amplitude or phase.

Method	Identified Number of People		Percentage	
	Amplitude	Phase	Amplitude	Phase
FCC	5	4	52%	43%
Our method	8	7	92%	85%
